# Crisis-line workers’ perspectives on AI in suicide prevention: a qualitative exploration of risk and opportunity

**DOI:** 10.1186/s12889-025-23298-8

**Published:** 2025-07-02

**Authors:** Jacob Greaves, Erminia Colucci

**Affiliations:** Middlesex University, London, UK

**Keywords:** Artificial intelligence, AI, Technology, Internet, Crisis, Suicide, Volunteers

## Abstract

**Background:**

Crisis support services offer crucial intervention for individuals in acute distress, providing timely access to trained volunteers whose human connection is key to the effectiveness of these services. However, there are significant disparities in who utilises these services. Recent advancements in artificial intelligence (AI) present new possibilities for crisis intervention in the form of AI-powered conversational partners. Yet, there is little guidance on how AI might be used in this context; and the risks and opportunities remain largely unexplored. This study aims to explore the risks and opportunities of integrating artificial volunteers (AVs) into crisis support, focusing on meeting the needs of those at risk of suicide. It collects the perspectives of crisis service volunteers to contemplate an AVs potential effect on the quality and effectiveness of support provided in crisis situations.

**Methods:**

A thematic analysis was used to interpret 13 semi-structured, exploratory qualitative interviews of an UK-based crisis service volunteers.

**Results:**

Three concerns were identified regarding perceived inflexibility and inauthenticity of AI, and the potential for dehumanisation of texters, aligning with factors influencing suicidal behaviour, such as perceived rejection and feelings of entrapment. Despite these concerns, the study found potential advantages, including reduced perceived burden on texters, enhanced confidentiality, and consistent, impartial responses. Recommendations for potential implementations suggest a cautious yet open-minded approach to integrating AI into crisis support, emphasising the need for transparency, accountability, and clarity on the role of human oversight.

**Conclusions:**

AI could complement rather than replace human support in the form of an AV, offering a differentiated, accessible avenue for those who might prefer or benefit from non-human interaction.

**Supplementary Information:**

The online version contains supplementary material available at 10.1186/s12889-025-23298-8.

## Introduction

Crisis support services are a vital component of the mental health care system in the United Kingdom, providing immediate intervention for individuals in acute distress or at risk of suicide. Traditionally, these services have relied on frontline volunteers trained in active listening to offer empathy and compassion as well as risk assessing, safety planning and de-escalation techniques [1–4]. While the evidence remains limited, the ability to share distressing emotions with another person is widely considered essential to the effectiveness of crisis services, as it plays a critical role in alleviating feelings of hopelessness, reducing psychological distress, and decreasing suicidality [5–6]. However, recent research suggests that artificial intelligence (AI) tools are increasingly being explored as an alternative to human support for mental health services [7–9]. This study examines the potential of AI as an alternative to human interaction during moments of crisis, specifically exploring how AI might serve as a conversational partner compared to human volunteers.

The integration of AI tools into healthcare has been largely driven by their potential to offer low-cost, scalable solutions that assist clinicians with routine tasks [10]. Within mental health care, text-based conversational agents have shown promise as accessible, user-facing tools that can supplement support for individuals experiencing anxiety, depression, and related issues [11]. The breakthrough of large language models, such as ChatGPT, have further accelerated this trend, prompting growing interest in their potential to contribute to conversation-based assessment and care [12]. A notable example is Limbic, a conversational AI therapy support tool which, when implemented in NHS talking therapies, was linked to greater treatment engagement, reduced dropout rates, and improved clinical outcomes compared to standard care [13].

While these tools show promise in supplementary mental health interventions, their use in higher-risk contexts has sparked concern. Critics argue that these technologies may lack the nuanced understanding required for effective care, dismissing them as mere “bullshit” machines [14]. Nonetheless, applications are beginning to be released and used in risk contexts, such as the suicide-specific Hope & Support Companion [15].

At time of writing, there is little to no guidance on the use of conversational AI in the suicidal context from authoritative UK mental health bodies or suicide focused stakeholders, i.e. NHS, BACP, Mind, Samaritans; and the same is true of APA in the US. The lack of guidance raises concerns that policymakers and developers might prioritise the perceived effectiveness, cost-efficiency and scalability of AI tools without fully considering their potential risks. Therefore, this study also aims to explore the risks and opportunities of using AI in crisis and suicide prevention services. Specifically, where someone in crisis can access a conversational AI-powered artificial volunteer (AV), rather than a human.

Stark experimental evidence of conversational AI tools shows that reliability for mental health support is poor, and decreases as cases get more severe [16] and is specifically unsuitable when assessing risk of suicidality [17–18]. Despite clear risks [9, 19], AVs may have a meaningful role to play in mental health support [20–21], with specific applicability in crisis services [22–23]. While robust evidence is still emerging, one early indicator can be seen in the substantial number of people engaging in conversations about suicidal ideation with AI chatbots, such as the ChatGPT-powered ‘Replika.’ [24].

One reason for this might be the stigma facing those with severe mental health struggles [25]. It is estimated that roughly half of those who die by suicide do not directly seek help nor openly disclose their intentions beforehand [26–27]. Even those in formal support systems often conceal or minimise their distress, driven by fear of judgment, shame, or concerns about losing their means to act [28–30]. These barriers appear particularly acute for some groups, such as men [28], who represent around one third of crisis service users [31–33], but account for approximately three quarters of suicide deaths in the UK [34].

In this context, the appeal of an AV may lie not in what it offers, but in what it lacks: the perceived judgment of another human. Research suggests people who believe they are speaking to a computer report lower fear of disclosing difficult thoughts and are more willing to open up emotionally [21]. It is within this landscape of silence, shame, and perceived risk that we explore how the presence or absence of a human in crisis support interactions may shape the experience and effectiveness of care.

The Integrated Motivational-Volitional (IMV) model [4] provides a theoretical framework to examine the interplay between the potential benefits of an AV and the critical aspects of human connection that may be compromised. The IMV model emphasises risk-moderating factors like connectedness, perceived rejection, and entrapment. This focus provides a level of specificity that may be lacking in other models, such as the Interpersonal Theory of Suicide [35]. This targeted approach allows for a nuanced exploration of how integrating an AV into crisis support could impact the effectiveness and potential risks associated with these interventions. By examining these specific factors, the IMV model offers a structured framework to assess both the potential benefits and drawbacks of transitioning from human to AI-driven engagement, ensuring a balanced evaluation of what is gained and what may be lost in this shift.

The aim of this research is to explore the potential of AI to deliver crisis response conversations, with a focus on how the absence of a human presence might affect the quality of support for individuals at risk of suicide. Rather than evaluating any specific AI technology, this study adopts a common-sense understanding of AI to examine what could be lost when human connection is removed from crisis interactions. Insights from frontline crisis service volunteers, who are deeply attuned to the relational and emotional dimensions of support, are central to this analysis. The study prioritises their perspectives and does not attempt a technical assessment of AI systems, which lies outside its scope.

## Methods

### Design

This study employed an inductively oriented reflexive thematic analysis of qualitative interviews to investigate the perspectives of volunteers at a crisis support service on the potential use of artificial volunteer (AV) by those in a crisis. The insights gained aim to inform plans for integrating AI more broadly into crisis support, highlighting both opportunities and potential challenges for service providers, software developers and policy makers.

Semi-structured interviews are well established as an effective data-collection tool for qualitative research by affording a researcher the capacity to adapt the focus of the interview to meet the beliefs offered by a participant, as well as to allow for probing when elaboration and clarification if needed [36]. The authors developed an interview guide [see Supplementary File 1] in which participants were asked about their views on AI in mental health care, whether they thought there might be any advantages or risks in using AI handling crisis conversations, and what recommendations they have for implementing an AI in the role of the volunteer.

The sample size was guided by the concept of information power (37), which holds that when the aim is narrow, the sample is highly relevant, and the data is rich, fewer participants are needed. Given the study’s relatively homogenous study population and focussed objectives, evidence suggests that a minimum sample of 10 participants would be sufficient to meet our objectives [38].

Reflexive thematic analysis [39] was chosen for its flexibility when comparing the firsthand experiences of seasoned crisis responders with their perspectives on how support might change if an AV were to attempt what they do, particularly in identifying ambiguous, subtle and potentially contradictory patterns and themes in the data. This approach allows for an in-depth exploration of the nuanced differences and imagined impacts, capturing the complex hypothetical trade-off between the value of human qualities and AI-driven responses in crisis settings.

The chosen setting for the research aimed to match existing AI capabilities with a suitable crisis service. The current generation of AI technologies primarily operate through a text interface. In this context, volunteers at a text-based service provide the most comparable baseline, minimising differences in communication medium that could affect effectiveness or functionality. In the UK, the predominant such service is Shout, a mental health text service run by Mental Health Innovations. Around a third of Shout text interactions involve suicide [40]. In addition to being a text service, Shout has developed an AI-driven simulation training tool in which volunteers interact with an artificial ‘texter in crisis’. This experience of artificial texters in crisis means that some participants will have an additional foundational aspect to discuss AI-powered crisis conversations.

### Participants

All Shout volunteers and staff with texter facing experience were given the opportunity to take part in the study. A final sample of 13 interviewees consisted of nine volunteers, two volunteer support (which involves volunteering and support for other volunteers), one clinician (a professionally qualified, paid texter facing position), one supervisor (responsible for guiding volunteers through high-risk procedures, such as safeguarding) and one trainer (responsible for developing and delivering training for volunteers). The average age was 46 years, and the average tenure was 3.6 years (range between 6 months and 6 years). There were 6 men and 7 women. All volunteers were UK-based, except one working remotely from New Zealand.

### Procedure

Participants were recruited through an advert posted on the charity’s internal online notice board and in the weekly internal newsletter. The advert outlined the aims of the study in brief terms and provided a hyperlink to a signup form in which they could read more about what would be involved and to obtain informed consent from all those who wished to take part.

Responses to the research advertisements were collected in an online form using SurveyMonkey. The form explained that the purpose of the study was to understand their perspective on AI technology used in crisis services, with a particular focus on suicide prevention, to understand the risks and opportunities of this technology. They were informed that the interview would last about 60 min and take place over video call. Their consent was attained for the interview to be recorded to enable later transcription, that what they said was confidential and that their data would be appropriately anonymised.

Throughout May 2024, 13 semi-structured individual interviews were conducted over video call to explore the perspectives of Shout listening volunteers, coaches and supervisors to understand the risks and opportunities associated with the potential future use of artificial volunteers in crisis services. Videocall was chosen instead of in-person interview for practical reasons, including the fact that the remote nature of Shout volunteering means that their team are not located in any one region. The content of the interviews was audio-recorded transcribed verbatim.

### Data analysis

The inductive-developed reflexive thematic analysis involves six phases: familiarisation with the data, coding key features, and searching for themes by collating related codes; focusing on patterns and themes that are most relevant to the research question [36, 39]. Themes are then reviewed and refined to ensure they accurately represent the data. Finally, themes are defined, named, and the analysis is reported in a coherent narrative of the findings.

The analytic process involved a thorough and iterative approach. After transcribing and re-reading each interview, and along with interview notes, a coding framework was devised and iterated as recurrent concepts were produced. An analysis table was populated with coded extracts and iteratively organised by participant and clusters of thematically related codes. After processing the data with initial codes, codes were clustered into themes in relation to broader psychological concepts, such as a sense of control. The coded data were analysed and weighted equally as a whole, and there was no attempt to analyse differences between types of participants, i.e. between volunteers and supervisors. Closely related subordinate themes were then further clustered and again assigned descriptive labels at a superordinate level. Preliminary themes shared in the form of a presentation to a group of participants and other stakeholders to gather feedback as a form of member checking results.

## Results

Table 1 shows the three superordinate themes, and nine subordinate themes, developed from the analysis of interviews with crisis hotline volunteers. *Concerns* reflect what an AV is expected to be lacking compared to the current human-based service, and what impact these limitations could have on texters. *Advantages* detail the potential advantages that an AV could offer in delivering crisis support and how it might positively affect texters’ perceptions and feelings toward the service. *Recommendations* reflect the challenges in implementing an AV, in terms of mitigating concerns and seizing opportunities in practice. Quotes have been pseudonymised and are attributed along with gender and role at Shout. All participants are given pseudonyms that reflect their gender to maintain confidentiality, there were no non-binary participants.

**Table 1:** Overview of themes and subordinate themes with single example quote

Themes and subthemes	Example quotes
Concerns	
Inflexibility of AI systems	“It feels like if things fit the model, then that could work. But what if it’s outside the model?” [Sue, Volunteer]
Inauthenticity stymies emotional safety	“If you’re trying to replicate the warmth, I think you probably could, but I don’t know. Then whether some level of just disingenuousness around that that then it would undercut it because it’s not a human being.” [Imogen, Volunteer] “I think it’s to do with the words that are chosen, I think it’s to do with the genuineness of the feeling behind those words.” [Sue, Volunteer]
Potential for dehumanisation	“They’ll say, ‘Oh, you’re giving up your time to hear me out’. I guess they know that someone’s literally sitting on the other end of the line.” [Jemima, Trainer] “People don’t feel they’re good enough to speak to a person.” [Karl, Volunteer]
Advantages	
Alleviating burden	“If you were talking with AI, you’re not burdening someone, you’re not burdening another real person.” [Edith, Volunteer]
Enhanced Confidentiality	“The whole confidentiality thing…more confidential than if they’re speaking to a real person?” [Edith, Volunteer] “And there is a fear out there that you’re gonna call the police. You’re gonna call the ambulance service. You’re gonna call my school.” [David, Volunteer]
Impartial and Consistent Support	“You can’t switch off that part of you that wants to make it better, whereas an AI could maybe sit more easily in that pain.” [Jemima, Trainer]
Recommendations	
Diversifying Support Options to Enhance Access	“If there’s a queue that’s four hours long and somebody disengages who’s at risk because they don’t get to talk to anybody, is it safer to have an AI that talks to them instead of having a person? You know, I’d argue probably yes.” [Karl, Volunteer] “There’s gonna be some that will go, ‘Actually, you know what? I’d rather stay with the computer right now.’” [David, Volunteer]
Needing Transparency	“As long as you’re upfront about what service you’re offering and what to expect. Then I think if people have the courage to give it a try, I think it could be very, very useful.” [Graham, Volunteer]
Establishing Accountability Mechanisms	“There’s got to be human safeguard in there somewhere.” [David, Volunteer]

### Concerns

Participants expressed a range of concerns regarding the integration of AI in crisis support, particularly focusing on the limitations of AI systems in handling complex and atypical interactions, the perceived lack of authenticity in AV responses, and the potential for an AV to contribute to a sense of dehumanisation among texters. These concerns highlight the critical role of human adaptability, genuine connection, and the value of presence in providing effective support, highlighting concerns about the essential elements that might be lost without a human volunteer.

### Inflexibility of AI systems

Participants expressed concerns about the potential limitations of AI in handling nuanced or complex interactions that deviate from standard models. They indicated that, while AI might be proficient in managing straightforward, routine conversations, its effectiveness could diminish in more complex scenarios. One volunteer noted:

*If you were struggling with anxiety*, *for example*, *and that might be*, *say*, *‘I’m being very anxious at the moment’. Then something might be able to guide you through a breathing exercise. You know*, *how about we work through this together and then see how you’re feeling after a few minutes?* [Graham, Volunteer Support].

This sentiment reflects a broader apprehension that an AV, bound by a predefined algorithm or training model, may lack the adaptability required to fully meet individual texter needs:

*It feels like if things fit the model*, *then that could work. But what if it’s outside the model? What if it’s somebody who finds it difficult to write*, *or to articulate*, *or to spell*, *or to find the language to explain how they’re feeling? I don’t know whether AI could do that. Possibly at some point in the future.* [Sue, Volunteer].

Participants discussed the importance of flexibility in their approach, often deviating from established protocols to better respond to texters’ unique situations. One volunteer articulated this adaptability stating: *“We talk about it quite a lot: you can have volunteers that don’t follow our model but get really good feedback*, *and you can have volunteers that follow our model perfectly and get terrible feedback.”* [Sally, Volunteer Support]. Such adaptability is perceived as essential in mental health support, where a rigid approach may lead to suboptimal outcomes. For example: *“You can fine tune the model […] But*, *you know*, *it’s mental health. You’re never gonna have a one-size-fits-all option for everybody. It’s just never gonna happen.”* [Karl, Volunteer]. Consequently, there is concern that AI, constrained by its programmed responses, may be less effective in addressing atypical or complex cases, particularly in the context of mental health where unpredictability is common: *“I think when you’re dealing with people’s emotions*, *something as complicated mental health*, *[it is] unpredictable sometimes*, *I think that’s where it could get a bit messy.”* [Edith, Volunteer].

### Inauthenticity stymies emotional safety

The importance of authenticity surfaced as a critical factor in building rapport and trust with texters. Participants underscored a sense of genuine empathy, care and compassion, builds the emotional safety needed for texters to feel safe in sharing their vulnerability. Lacking a mechanism for building emotional safety, participants question whether an AV would be able to provide effective support in crisis situations. One volunteer emphasised:

*If it’s a robot telling you [something]*, *I think you can kind of know the robot has just been programmed to say that it*, *[it] doesn’t actually believe it. And I think it might be important for texters to feel like there is a person who actually believes what they’re saying.* [Hailey, Volunteer].

Participants highlighted that their training equips them to convey genuine care through carefully chosen words, which can foster a sense of connection and trust even in text-based communications. However, without the feeling behind the words, they might lack the same weight. As one volunteer pointed out:

*Psychologists who said it is not possible to demonstrate empathy online*, *and I disagree*, *so I suppose how is it I demonstrate empathy online? I think it’s to do with the words that are chosen*, *I think it’s to do with the genuineness of the feeling behind those words.* [Sue, Volunteer].

Additionally, a sense of authentic connection may play other crucial roles during a crisis, such as providing texters with a reciprocity-based reason to keep living:

*You get a sense in many people that they don’t want to hurt somebody else. And so*, *if there’s a person at the other end of the text or call*, *then going through it*, *most people - I get a sense - would see that as like letting that person down. They don’t care about themselves*, *but they might care about somebody else. And if they’ve made that connection with somebody else*, *they care about that. I think it’s difficult to get that from a computer.* [David, Volunteer].

### Potential for dehumanisation

Beyond issues of authenticity, there were concerns that an AV could contribute to a sense of dehumanisation among texters, in the sense that it could contribute to feelings of worthlessness and rejection. The concern was partly inherent in the perception of AI, and chatbots more broadly, as a cost-saving substitute for human interaction, potentially exacerbating feelings of worthlessness or rejection among texters. As one volunteer noted:

*You know if somebody else [contacts Shout] and they get a person*, *‘Why are they getting a person and I’m getting a bot?’ and whether that might go back to the sort thing [I] mentioned where people don’t feel they’re good enough to speak to a person.* [Karl, Volunteer].

Another part is that participants believed that their presence conveys to texters that they are valued and heard, which is central to the support they provide. One volunteer underscored the value of presence by stating:

*If they know it’s a chatbot*, *they know very well there is no person who is listening to them*, *and I feel like that could make them feel like there’s no person in the world who actually cares about them.* [Hailey, Volunteer].

Presence was linked to feeling valued in the follow way:

*And a lot of the texters will say like ‘Nobody cares.’ So I think maybe it is about feeling heard by someone*, *‘cause like feeling heard allows you to feel connected because you’re like*, *‘Wow*, *this person’s listening to me’. There’s someone who does care. Like often they’ll say*, *‘Oh*, *you’re giving up your time to hear me out’. I guess they know that someone’s literally sitting on the other end of the line. Which it could be at like 3:00 AM […] giving them the time.* [Jemima, Trainer].

Another volunteer illustrated the importance of simply feeling heard with the following: *“You explore what’s going on for them and you’re trying to get to the goal and*, *actually*, *the goal is just be heard. And they say*, *‘Right*, *OK*, *well*, *I made my cuppa. Thanks for the chat. I’ll speak to you tomorrow.’”* [Sally, Volunteer Support]. In contrast, interactions with AI may lack this personal touch, leading texters to feel that they are engaging with a faceless, impersonal entity rather than a caring individual. For example: *“It’s almost like an extension of*, *‘Press 1 for this*, *Press 2 for that*, *Press 0 to wait for a real person kind of thing.”* [Graham, Volunteer Support].

### Advantages

Participants identified three key advantages of using AI integration to enhance crisis support, focusing on its potential to alleviate feelings of burden among texters, enhance confidentiality, and provide impartial, consistent support. These advantages highlight how an AV can complement existing services by addressing specific psychological barriers faced by individuals in crisis, such as fears of judgment, concerns about confidentiality, and the variability of human responses.

### Alleviating burden

Participants identified that one of the key advantages of AI in crisis support is its potential to reduce the sense of burden that texters may feel when seeking help. At the same time, many texters express to volunteers that they worry about imposing on others, including volunteers, with their problems. Participants understanding is that this concern often leads them to hesitate or avoid reaching out altogether. As one volunteer explained, texters sometimes apologise to volunteers for sharing their struggles, reflecting their anxiety about being a burden:

*Almost that notion of: if you were talking with AI you’re not burdening someone*, *you’re not burdening another real person. Very often you have texters say*, *‘I’m really sorry you’re having to listen to all this’. And you know*, *they apologise*, *and I [say]*, *‘Nothing to apologise for. That’s what I’m here for*,*’ or whatever. But I could see how for some people that might alleviate some of that sense of burden.* [Imogen, Volunteer].

The sense of burden is commonplace for individuals contacting crisis services, who often feel isolated or disconnected. Many texters express feelings of loneliness and worry about burdening friends, family, or even volunteers. As one volunteer observed:

*They feel lonely and we [have] got so many people that say*, *‘I’ve got no one to talk to’ and that’s their depression or their anxiety talking. And actually*, *when it comes to it*, *they do have people to talk to. They’ve just chosen not to*, *or they feel like they don’t wanna be a burden on people.* [Sally, Volunteer Support].

For these texters, an AV offers a unique form of social safety in conversation, providing a space where they can share their emotions without the need to face human interaction. One volunteer said: *“[It’s like] talking into the void a little bit. Like you’re getting*, *you’re getting your emotions out in some way without the pressure of knowing there’s another person listening.* [Edith, Volunteer].

Counter to above-mentioned importance of human connection, this sense of distance or social safety could make it easier for them to initiate contact with the service, as it allows them to share their experiences with freedom from the perceived judgment or emotional burden associated with human interaction. One volunteer suggested: *“I think it’s a way potentially for people to open up when they feel unable to open up to a human.”* [Hailey, Volunteer]. Another volunteer expressed the complex and often ambivalent emotions that texters might experience when seeking help from another person:

*For them it might [feel like] actually no support in the world is going to make any difference. But more likely*, *it’s actually at that point they don’t want to be talked out of it. Because they believe that if they talk to a person*, *that person will find a way of getting into a place where they don’t go through with it.* [David, M, Volunteer].

This sense of safety can be especially crucial for individuals who have experienced trauma or abuse. AI provides a non-judgmental, anonymous platform that may feel less threatening than human contact. One volunteer explained:

*People who have experienced sexual abuse*, *they feel a lot of shame even talking about it. So*, *I wonder if maybe AI could help them there because they get to share it*, *but they know that no one is judging them or blaming them for what happened. Sometimes those conversations with abuse*, *you know*, *you might get so far*, *and then they just disengage because it’s too much or they feel really vulnerable again or like they don’t want to talk to a male volunteer.* [Jemima, Trainer].

This highlights AI’s potential to facilitate first contact for those who may be reluctant to engage with human support, offering a sense of control and distance that can be vital for their willingness to seek help.

### Enhanced confidentiality

Participants also identified enhanced confidentiality as a significant advantage of AI in crisis support settings. Participants reported that many highly vulnerable users abruptly end conversations when asked for identifying information:

*In a high-risk situation*, *where we believe [it] is building towards one of these [emergency service interventions]*, *then we will start asking more and more intrusive questions; ‘What’s your surname? Where do you live? Can I have a post code? Do you know where exactly you are?’ I would say*, *I pick a number. I can’t guarantee this*, *so you can’t use it as a piece of data*, *but I would say probably 75% of the time [texters stop the conversation] at that point.* [Tim, Volunteer].

They noted that some texters struggle with the stigma associated with seeking help, particularly regarding people in their lives finding out about what they have shared. One volunteer observed: *“There is a fear out there that you’re gonna call the police. You’re gonna call the ambulance service. You’re gonna call my school.”* [David, Volunteer]. In this context, an AV could offer a safer and less intimidating entry point for individuals hesitant to engage with existing channels, for example:

*I do know*, *like some people*, *I think they’re their main concern with texting a service like Shout or calling Samaritans is*, *‘Oh*, *they’re going to call the police on me’. So if they thought that the chatbot would not do that*, *perhaps that would be an appeal for them.* [Hailey, Volunteer].

Another volunteer raised it as a question, indicating uncertainty about whether all texters would experience the same enhanced sense of confidentiality:

*I suspect some people might be happily speaking to an AI*, *you know*, *the whole confidentiality thing. If you’re speaking to AI*, *would a texter feel that their confidentiality is more confidential than if they’re speaking to a real person?* [Edith, Volunteer].

Overall, participants’ responses suggest that an AV has the potential to play a role in facilitating access to support by addressing texters’ privacy concerns more effectively than human-based services.

### Impartial and consistent support

Another advantage identified was the potential for AI to provide impartial and consistent support, particularly in high-stakes situations where human bias might inadvertently affect judgment. With just a few words of text as their data, volunteers are required to manage multiple complex tasks, including assessing risk. Most participants explained the value of intuition in anticipating risk or understanding texter’s needs, for example:

*There is more unsaid than is actually said by the texters and you can you can get a real impression of them*, *just even by the speed with which they reply*, *by the way they reply and I don’t know that AI would be sufficiently intuitive to pick that up […] You do have texters who you can hear them thinking about what they’re what they’re going to say*, *that you can actually*, *I can hear them*, *you know*, *or I can visualise them sitting there making a decision about*, *‘Am I gonna say this?’ or ‘Aren’t I going to say this?’ which AI wouldn’t pick up*, *I don’t think.* [Edith, Volunteer].

However, some also acknowledged that, while human intuition is valuable, it can also lead to inconsistencies in how texters are supported, for example:

*Because you’re dealing with text*, *I think your mind wants to fill in gaps. Humans in lots of places will read into things*, *rightly or wrongly. I suppose you might try and build up the picture if you don’t have all the facts kind of thing you might read into things. Does that help? Or hinder?* [Graham, Volunteer Support].

Overreliance on intuition, rather than following best practices, can lead to misjudgements, missed risk, and misconceptions about what a texter needs. A Volunteer Support participant suggested:

*I think the biggest thing is*, *and I don’t see an issue with AI doing this. A lot of the [volunteers] kind of assume or they think they know what the texter wants and*, *actually*, *they haven’t asked them. That’s kind of where the conversation can go wrong a lot. […] You kind of know or hope that an AI is going to ask the question every time*, *whereas I know from looking at conversations that volunteers take they often presume that [they] know what the texter wants and they actually haven’t asked them.* [Sally, Volunteer Support].

Moreover, a trainer suggested that one prevalent deviation from best practice might be inherent to human nature, implying that an AV’s impartiality to the texter could enable it to perform better than a volunteer in this type of situation:

*One of the things that I guess is a limitation of volunteers*, *is sometimes human nature. You kind of wanna help someone*, *right? You don’t want them to be feeling pain*, *and a lot of our volunteers can be drawn into trying to make it better. And you can’t switch off that part of you that wants to make it better*, *whereas an AI could maybe sit more easily in that pain.* [Jemima, Trainer].

This impartiality could foster a supportive environment where texters feel validated and understood without the complications of human subjectivity, making AI a potentially valuable complement to existing services.

### Recommendations

Trying to reconcile the concerns and advantages identified, participants’ recommendations for a potential AV integration emphasise the need to diversify support options, maintain transparency, and establish robust accountability mechanisms. They suggested that an AV could complement human support by providing an option for texters who prefer - or would only seek help through - an AV, thereby relieving some of the strain on human resources while ensuring that diverse needs are met. Participants also highlighted the importance of being transparent with texters to build trust and set realistic expectations. Furthermore, they underscored the critical need for accountability measures to ensure that the AV is safely and ethically managed, particularly in high-risk situations, thus preserving the integrity of crisis intervention services.

### Diversifying support options to enhance access

Participants acknowledged the current limitations of their service, particularly the strain on human resources and the diverse needs of individuals in crisis. They suggested that integrating AI as a supplementary service could address these challenges without replacing existing human support. Participants emphasised that AI could alleviate some of the pressure on human resources by providing an option for texters who prefer to interact with AI, thereby allowing human support to be reserved for those who seek personal interaction. One volunteer expressed their distress over the growing demand for crisis support and the resulting strain on limited resources, highlighting how these pressures can currently manifest:

*Arguably if there’s a queue that’s four hours long and somebody disengages who’s at risk because they don’t get to talk to anybody*, *is it safer to have an AI that talks to them instead? You know*, *I’d argue probably yes.* [Karl, Volunteer].

Others stress the need for fast responses from the service: *“It might even be able to take enough that you don’t even have a waiting list. And people can get an immediate response*, *which sometimes when they’re in a crisis state*, *I think is really necessary.”* [Hailey, Volunteer].

By offering both AV and human options, the service could better cater to the varied preferences of individuals in need, ensuring that support is available in a form that feels most comfortable and accessible to each texter. As one volunteer remarked:

*It’s difficult to put yourself in somebody else’s shoes in that sense. I know I wouldn’t [rather talk to the AI]*, *but almost by logic some people would. If we just [look] in a normal statistical distribution of people*, *there’s gonna be some that will go*, *‘Actually*, *you know what? I’d rather stay with the computer right now.’* [David, Volunteer].

At the same time, participants understand that an AV will not be the preferred choice for all users. Therefore, they believe that any AI service should supplement, not replace, existing human services:

*I suspect that there’s always going to be a need for human contact in some way and to be it I would hope*, *but I don’t know. There’s always a difference between the human contact and the computer generated one […] I don’t see it as things stand […] as being a replacement.* [John, Volunteer].

This reflects the broader sentiment that diversifying support options could significantly enhance the reach and effectiveness of crisis intervention services. One volunteer’s remarks particularly underscore the strong sentiment around seizing opportunities to address limitations:

*Shout should be looking at it because surely*, *it’s got to be something*, *you know*, *we know that our queues are too big*, *we know that we don’t have enough volunteers. So why are we not looking at every opportunity to make life better for the person texting? So*, *if they turn around and said we’re not looking at it*, *I’d be surprised.* [Tim, Volunteer].

### Needing transparency

Another key recommendation was the importance of transparency regarding the nature of AI interactions. Participants emphasised that texters should be fully informed about whether they are interacting with a human or AI, as this transparency could foster trust and empower users to make informed choices about their support. One volunteer stated:

*They’ve been able to make a decision and a choice*, *and they’ve got the control and the choice to do that. And that is as important as if they didn’t text stop and we were able to continue the conversation that they*, *they can choose to disengage.* [Imogen, Volunteer].

This highlights the ethical imperative to provide clear and honest communication to texters about the role of AI in their support experience.

Participants also suggested that transparency could help set appropriate expectations and mitigate potential frustrations that may arise from the limitations of AI. As one volunteer pointed out: *“You’d probably be more forgiving. You probably kind of go*, *‘I understand these aspects because it’s not a real person’. But I still think you might be able to accept the process and just work with it.”* [James, Clinician]. Participants’ expectations that texters might accept AI support likely stem from the fact that nearly all volunteers have, at some point, been asked if they were an AI themselves. As a Volunteer Support noted:

*You get a lot of references to*, *‘You sound like a robot. Are you a robot?’ Especially when they’re kind of newer volunteers and they’re kind of finding their feet*, *the texter [might] say*, *‘Are you robot or your computer? Are you AI?’* [Sally, Volunteer].

This approach not only respects the autonomy of texters but also help them understand what they can expect from interacting with an AV. As one volunteer noted:

*As long as you’re upfront about what service you’re offering and what to expect. Then I think if people have the courage to give it a try*, *I think it could be very*, *very useful. You know*, *I’m all about human connection*, *but I think at the same time*, *there are certain aspects where it would really help.* [Graham, Volunteer Support].

### Establishing accountability mechanisms

Transparency alone, though, is not enough. Participants underscored the need for robust accountability mechanisms to accompany the integration of AI into crisis support services. A central concern for volunteers is their responsibility to keep texters safe. Acknowledging the challenges in doing so, and the reality that some texters may still come to harm after using the service, volunteers rely on support from supervisors and clinical experts. One volunteer illustrates this support and sense of responsibility:

*The way our system is*, *if a mistake is made by a volunteer*, *and especially with where I’m at because I’m quite early on still*, *I’m still quite reliant on supervisor support. So*, *there is a team of clinical supervisors and often they might pick up on something that a volunteer has missed*, *so there’s another layer of checks.* [Imogen, Volunteer].

Participants expressed concerns about the potential risks associated with an unsupervised AV, particularly in situations where texters may be at acute risk of harm. One volunteer reflected:

*There’s got to be human safeguard in there somewhere. Somewhere there needs to be inbuilt flags*, *I think*, *for the more serious [cases] to allow to allow a human to go in and go*, *‘Is this actually doing what we want it to do?’* [David, Volunteer].

This recommendation points to a sense of necessity for human oversight in critical decision-making processes.

Additionally, participants highlighted the importance of developing clear guidelines and protocols for an AV use, particularly around sensitive procedures like safeguarding and emergency service interventions. As one volunteer suggested: *“I guess it depends if there’s still supervisors making the clinical decisions. I don’t think it’s black and white of when you need to call in an active rescue or a safeguarding call.”* [Jemima, Trainer]. These measures would help to establish a framework of accountability, ensuring that AV remains a tool that supports, rather than undermines, the core responsibilities of crisis intervention services.

## Discussion

The primary aim of this study was to explore the potential of AI-powered artificial volunteers (AVs) as an alternative to human interaction in crisis support services, specifically examining how AVs might impact the quality and effectiveness of support provided to individuals in distress. By focusing on the perspectives of experienced crisis service volunteers and those that support their work, the study sought to understand the potential risks and opportunities associated with integrating AI into crisis interventions. Earlier studies primarily focus on AI’s general capabilities, such as delivering empathetic responses [41]. This research delves deeper into how these systems interact with individuals at their most vulnerable moments, particularly within the framework of the Integrated Motivational-Volitional (IMV) model [4]. This differentiation underscores the contribution this study can make to the discourse on AI in high-risk mental health contexts.

Key findings revealed three major concerns regarding the use of AVs in crisis support: inflexibility, inauthenticity, and dehumanisation. These concerns align with the IMV model highlighting factors such as perceived rejection, lack of connectedness, and feelings of entrapment, which are critical in influencing suicidal behaviour. Despite these concerns, the study also identified potential advantages of AI, such as reducing the perceived burden on texters, enhancing confidentiality, and offering consistent and impartial responses. These findings underscore the need for a balanced approach to integrating AI, ensuring that the benefits do not come at the expense of the essential human elements of crisis support.

### Concerns

The concern of inflexibility underscores AV’s limited capacity to respond to the nuanced and unique needs of individuals in crisis, which could exacerbate feelings of entrapment, a key factor in suicidal behaviour according to the IMV model. Unlike human volunteers who can adapt their approach based on real-time cues and individual differences, AI systems are limited by the logical constraints of their training data [42]. This rigidity could limit the AI’s effectiveness in handling complex or atypical cases, such as those described by Elyoseph and Levkovich [17], potentially failing to intervene successfully in the critical progression from suicidal ideation to intent. The lack of adaptability in AI may leave texters feeling hopeless or alone, amplifying their sense of being trapped or unheard in their specific struggles [43]. The theme of inauthenticity relates to the potential lack of mechanism for building genuine social connection in AV interactions. Within the IMV framework, social connection is crucial in mitigating feelings of rejection and thwarted belongingness, both of which are significant predictors of suicidal ideation [44]. Studies show that AI can reliably deliver empathetic messages [41]. However, the AV’s lack of humanity may render this empathy ineffective, potentially heightening perceived social disconnection. Texters might feel that responses are formulaic rather than genuinely supportive [45]. This raises questions about whether an AV could sufficiently fulfil the role of crisis hotlines, where the opportunity to safely share thoughts and feelings provided by volunteers serves to help regulate their emotions. Relatedly, the potential for dehumanisation focuses on the potential for an AV to subtly reinforce feelings of rejection, which the IMV model emphasises as moderating factors of suicidal behaviour [46]. Rather than a caring individual demonstrating willingness to spend time listening to them, volunteers worry that texters who are already sensitive to rejection may feel they are only able to interact with a machine due to resource constraints. In turn, risking exacerbating damaging feelings of burdensomeness and stigmatisation, and therefore suicidality [47–49].

While participants’ concerns about the inauthenticity and dehumanising effects of AI are well-grounded, it is also important to consider the potential biases that may shape these views. Participants, as human crisis responders, may inherently perceive AI as a threat to the roles they currently fulfil, which could predispose them to be more critical of AI systems. This reaction is not uncommon when technological interventions disrupt traditional human roles [50]. Additionally, AI itself is not free from bias. Algorithmic models can reflect societal inequalities, potentially leading to disparities in risk assessments for different demographic groups. A deeper examination of both human and algorithmic biases can help contextualise the volunteers’ concerns, recognising that their scepticism may stem as much from perceived challenges to their vocation or expertise as from the limitations of AI itself.

### Advantages

Despite these concerns, the themes of alleviating burden and enhanced confidentiality could work to lower the threshold for individuals to seek help and encouraging more open disclosure of suicidal thoughts. While participants raised concerns about an AV being dehumanising, the theme of alleviating burden posits that freedom from social interaction may offer relief from the barrier of feeling like a burden and other hurdles facing underserved groups. The barriers detailed in recent work by Bennett and collaborators [51] are normative social pressures, feeling unworthy of resource, stigma, fear of judgement, and inability to describe emotions. In this context, non-human conversational agents are often viewed as more acceptable by individuals when dealing with issues that carry a higher level of stigma [52], or if potential users feel that their struggles are not serious enough to seek help. By lowering the height of a first step these benefits an AI service might extend the reach to previously unserved groups.

Lowering these specific barriers to help-seeking may impact not just who uses the service, but also how it is used by texters. A service with lower psychological stakes can invite use before the point of crisis that may improve texter mental health literacy [53] and inform texters about what types of help exists [54], which may improve help-seeking and care utilisation if the point of crisis arises [53].

Relatedly, an enhanced sense of confidentiality may provide a sense of protection for potential users who fear friends and family knowing what they have shared or fear the police or school might act to take away the means to act harmfully towards themselves [55]. However, in the UK, services like Shout have a statutory duty to safeguard children and vulnerable adults. While the capability for a highly confidential ‘sandboxed’ conversation with an AV may be theoretically possible and appealing, it may be that there are legal barriers that prohibit operating in this way.

The opportunity for enhanced impartiality is distinct in that it addresses an arguable limitation of human volunteers. The ability to recognise patterns and make quick judgements with highly limited information can lead to bias, misjudgement and overconfidence in decisions [56, 57]. The personal experience and contexts of volunteers can influence judgements about what a service user needs and wants. Another perspective comes from the concept of the ‘nocebo’ effect [58], which highlights how negative expectations and communication can inadvertently harm patients by validating distressing beliefs or feelings. AI’s ability to consistently maintain a calm and neutral tone may aid in delivering supportive messages. This could reduce the variability seen in human interactions, creating a more predictable and secure environment for users. Thus, while AI cannot replicate genuine human empathy and compassion as suggested by its inauthenticity, its consistency and neutrality could serve as a buffer against the inadvertent nocebo effects that human interactions might sometimes produce, thereby offering a distinct form of emotional safety [58]. The result could be greater adherence to best practice and consistency of service.

### Recommendations

Participants’ recommendations centre on how AI-based crisis services could be effectively integrated while respecting the strengths of human volunteers. By including AI as an additional option for texters, an AV could fill gaps in current crisis support by offering a different option for those who might feel more comfortable engaging with technology rather than a human. Diversifying support options to enhance access suggests that the value of an AI service lies not in replacing human support but in complementing it. As a standalone intervention, AVs could provide a valuable alternative that meets users at their comfort level, akin to self-guided interventions, which are shown to be effective suicide prevention interventions particularly for those with limited access to help [59]. Simultaneously, if an AV is preferred by a portion of current texters and they elect to use that alternative service, limited volunteer resources can be more readily utilised by texters who do want to talk to a human. A consequence of this might mean shorter waiting times for those wanting, or more urgently needing, a human conversation, but also greater symmetry in matching their needs and the volunteers’ skillset.

Transparency about the nature of an AV would help ensure that texters are fully informed that they are interacting with a machine, not a person. Ethical AI guidelines generally regard transparency as essential for maintaining trust and for setting realistic expectations about the type of support that can be provided [60]. Transparency empowers texters with the choice to engage with AI knowingly, fostering a sense of agency and control, which the IMV suggests can be valuable first steps for those in acute suicidal distress [4].

The need for accountability mechanisms was a significant concern among participants, particularly regarding the safety of texters. Shout supports volunteers with a network of supervisors and clinical experts to guide decisions with urgent and high-risk ethical compromises, such as emergency service interventions. How well an unsupervised AI system can weigh ethical decisions in a crisis is questionable [61], especially when dealing with sensitive issues such as suicidality. To address these concerns, participants recommend that AI systems should include mechanisms for human supervision, particularly for decisions involving high-risk texters. While there is agreement on the need for an oversight model, the ideal approach remains uncertain; for example, further research is needed to determine whether crisis AI applications might involve human-in-the-loop monitoring, periodic expert review, or another model entirely [22].

However, transparency about human oversight of an AV might well negate all the advantages of greater confidentially, impartiality, and to a lesser extent the freedom from burdensomeness. Balancing oversight challenges with promise of confidentiality is open and represents something of an *automation conundrum*. That is; as a system is given greater autonomy, while its reliability and robustness increase, there is less opportunity for oversight or intervention by human operators [61].

Beyond the practical recommendations for transparency and supervision, the ethical implications of integrating AI into crisis services warrant further exploration. Crisis intervention is a high-stakes environment where decisions can significantly impact texters’ safety. The ethical risks of relying on AI, particularly in instances where human lives are at stake, such as suicide prevention, raise critical concerns. AI errors, no matter how rare, could result in inadequate responses during a crisis, exacerbating rather than alleviating distress. These risks highlight the need for robust ethical frameworks tailored specifically to AI’s role in sensitive mental health interventions.

While traditional crisis hotline models have been built around human-to-human interaction, the emergence of AVs suggests that these models may need to evolve to reflect the potential benefits that AI can bring to crisis intervention. Currently, crisis hotlines operate under the assumption that human volunteers are best suited to handle the emotional and psychological needs of individuals in crisis. However, our findings suggest that AVs offer specific advantages, such as impartiality, enhanced confidentiality, and the ability to consistently deliver supportive responses, which could be especially useful for individuals hesitant to reach out to human volunteers.

### Implications for future research and practice

Future research that involves potential users of an AV is needed to add depth of understanding to the ideas explored in this research. This might include further qualitative exploration of the potential user groups, such as confidentially minded adolescents or men struggling to take steps to find help. Whereas experimental or trial evidence could provide grounded insights into texters experience of using an AV.

Another critical aspect of AI integration in crisis support that warrants attention is its potential to shift power dynamics and impact texters’ sense of agency. The introduction of artificial volunteers may change how texters perceive their own control over the support they receive. While AI can offer impartial and consistent responses, it may also create a dynamic where texters feel more distanced from decision-making processes. The ability to choose between AI or human support is an important consideration for maintaining agency. How AI shifts this relationship, whether texters feel more empowered by the anonymity or less connected due to the absence of human interaction, should be a key area of future research.

Other future research could explore the implications of AI integration on organisational risk management. For example, investigating how communication about AI’s role in crisis services impacts the trust of stakeholders and those who donate to charities would be valuable. This could include examining strategies for explaining AI’s capabilities and limitations to funding bodies in ways that align with ethical standards and organisational values, minimising potential backlash or loss of support.

As AI systems are increasingly adopted in sensitive areas like mental health support, research could focus on developing comprehensive ethical and legal compliance frameworks specifically tailored for AI in crisis services. These frameworks would guide organisations like Shout in navigating the complex landscape of legal obligations, including data protection, user safety, and mandatory reporting. Research could investigate how these frameworks can be standardised across the industry to ensure consistent application and adherence to best practices.

### Limitations

The study is grounded in the perspectives of hotline volunteers, focusing on their expertise in delivering crisis response. Consequently, the findings reflect concerns and considerations related to the delivery aspects of crisis intervention, rather than from the perspective of users or those with technical AI expertise. Future research could benefit from integrating technical assessments alongside user-centred insights to provide a more holistic understanding of AI’s role in crisis support and how it might psychologically and behaviourally affect those that use such a tool.

The study design leaves the results with significant biases. The sampling strategy is biased towards crisis response volunteers with a particular interest in the subject of AI, which may have skewed the results. Moreover, at an organisational level, more long-established services like Samaritans may have a greater sense of caution regarding the idea of an AV, possibly meaning Shout are not representative of other major crisis services and this research should be repeated with other services. Also, as AI-based crisis support services increase and so does the pool of users, research should explore the socio-demographics of those more likely to successfully engage with such services and highlight some potential patterns, such as AVs being more appealing to higher socioeconomic status and level of education, and less to ethnic minorities and non-native English speakers.

## Conclusion

Overall, the findings highlight ambivalence toward the prospect of introducing an AV into crisis support. Aligned with core concepts of the IMV model, the introduction of an AV may conceivably either increase or reduce feelings of burdensomeness, stigmatisations or thwarted belongingness. Nonetheless, the recommendations suggest a cautious but open-minded approach to integrating AV. On one hand, there are clear concerns that an AV would lose critical aspects of what make the current service effective. On the other hand, there are opportunities to lower barriers to help-seeking and improve the service where there are currently issues. Participants see value in diversifying the ways in which support can be accessed but emphasise the importance of transparency and accountability to ensure that AI can enhance, rather than undermine, the support currently provided by human volunteers. By addressing these key areas, AI-enhanced crisis services could better align with the values and needs of those in crisis, offering an accessible and valuable extension of existing support systems.

## Electronic supplementary material

Below is the link to the electronic supplementary material.

Supplementary Material 1

## Data Availability

The corresponding author would consider requests to access anonymised data.

## Abbreviations

AI: Artificial Intelligence
AV: Artificial Volunteer
IMV: Integrated Motivational-Volitional
