# Supplementary material for: Crisis-line workers’ perspectives on AI in suicide prevention: a qualitative exploration of risk and opportunity
Source: BMC Public Health. 2025 Jul 2;25:2229. doi: 10.1186/s12889-025-23298-8 (PMC12220356; doi:10.1186/s12889-025-23298-8)
Supplement: Supplementary file 1 — Supplementary Material 1 [file 12889_2025_23298_MOESM1_ESM.docx]

Discussion guide

Total Duration: Approximately 60 minutes

Introduction (5 minutes)

- Explain who I am.
- Check their details.
- Explain the format of the interview.
- Explain the purpose of the interview and how the data will be used.
- Confirm participant has read the consent form and agrees to proceed.
- Reassure about confidentiality and the right to withdraw at any time.

Background and Experience (10 minutes)

- Can you tell me about your role as a volunteer/trainer at Shout?

Perspectives on AI in crisis support (15 minutes)

- Can you tell me what you know about the use of AI technology/chatbots, like ChatGPT, in mental health and crisis services?
  - Introduce AI and GPT technology if necessary
- Have you had any direct experiences with AI tools or technologies in your role?

Possible Advantages (10 minutes)

- Can you think of any advantages that AIs might offer in crisis services?
  - And with suicide prevention specifically?
- Do you believe there are specific situations where AIs might be particularly helpful?
  - Probe on any mention of ‘who’ might benefit

Concerns and Risks (10 minutes)

- What concerns do you have about AI being used in crisis services?
- How do you feel about AI handling suicidal conversations?

Recommendations and Final Thoughts (5 minutes)

- Practically, how do you envision AI could be used by services like Shout?
  - Clarify that this is not about Shout doing this, but crisis and suicide prevention services.
- What recommendations would you have for an ethical implementation of AI in this context?
- Do you have any final thoughts or anything else you would like to add?

Conclusion and Debrief

- Thank the participant for their time.
- Use debriefing document to;
  - Outline the next steps and how they can access the results of the study.
  - Provide contact information for any follow-up questions or support.
